# Mtss1 Promotes Cell-Cell Junction Assembly and Stability through the Small GTPase Rac1

**DOI:** 10.1371/journal.pone.0031141

**Published:** 2012-03-27

**Authors:** John C. Dawson, Susann Bruche, Heather J. Spence, Vania M. M. Braga, Laura M. Machesky

**Affiliations:** 1 Beatson Institute for Cancer Research, Glasgow, United Kingdom; 2 Faculty of Medicine, National Heart and Lung Institute, Imperial College London, London, United Kingdom; University of Birmingham, United Kingdom

## Abstract

Cell-cell junctions are an integral part of epithelia and are often disrupted in cancer cells during epithelial-to-mesenchymal transition (EMT), which is a main driver of metastatic spread. We show here that Metastasis suppressor-1 (Mtss1; Missing in Metastasis, MIM), a member of the IMD-family of proteins, inhibits cell-cell junction disassembly in wound healing or HGF-induced scatter assays by enhancing cell-cell junction strength. Mtss1 not only makes cells more resistant to cell-cell junction disassembly, but also accelerates the kinetics of adherens junction assembly. Mtss1 drives enhanced junction formation specifically by elevating Rac-GTP. Lastly, we show that Mtss1 depletion reduces recruitment of F-actin at cell-cell junctions. We thus propose that Mtss1 promotes Rac1 activation and actin recruitment driving junction maintenance. We suggest that the observed loss of Mtss1 in cancers may compromise junction stability and thus promote EMT and metastasis.

## Introduction

E-cadherin is the major epithelial cadherin and it is frequently lost during epithelial to mesenchymal transition (EMT [1]) and cancer metastasis. Cadherins link adherens junctions to the actin cytoskeleton [2]. The small GTPase Rac1 is a key regulator of the epithelial actin cytoskeleton, which influences dynamics of cell-cell contacts [3], [4], [5], [6], [7]. Rac1 is activated upon E-cadherin clustering during de novo cell junction formation and activity decreases as junctions mature [4], [7], [8]. Activation of Rac1 inhibits the constitutive endocytosis of E-cadherin via recruitment of IQGAP-1 and F-actin to cell-cell junctions [9], [10].

Metastasis suppressor-1 (Mtss1) is a member of the IMD-family (IRSp53 and MIM domain) [11]. Mtss1 is expressed in early phases of tumorigenesis but is lost in metastatic cells and is thus a putative metastatic suppressor thought to inhibit cell motility [12], [13], [14], [15], [16]. Mtss1 is required for maintenance of intracellular junctional integrity in the mouse kidney and co-localizes with E-cadherin in MDCK cells where it promotes F-actin assembly [17]. Mtss1 is also required for border cell migration in *Drosophila* oocytes [18], which migrate between adjacent nurse cells using *Drosophila* E-cadherin [19]. Mtss1 induces Rac1, but not Cdc42, activation via the IMD but not directly as a Rac1-GEF [20], [21], [22], [23].

## Results and Discussion

### Mtss1 inhibits HGF-induced cell scattering

**Figure 1:**
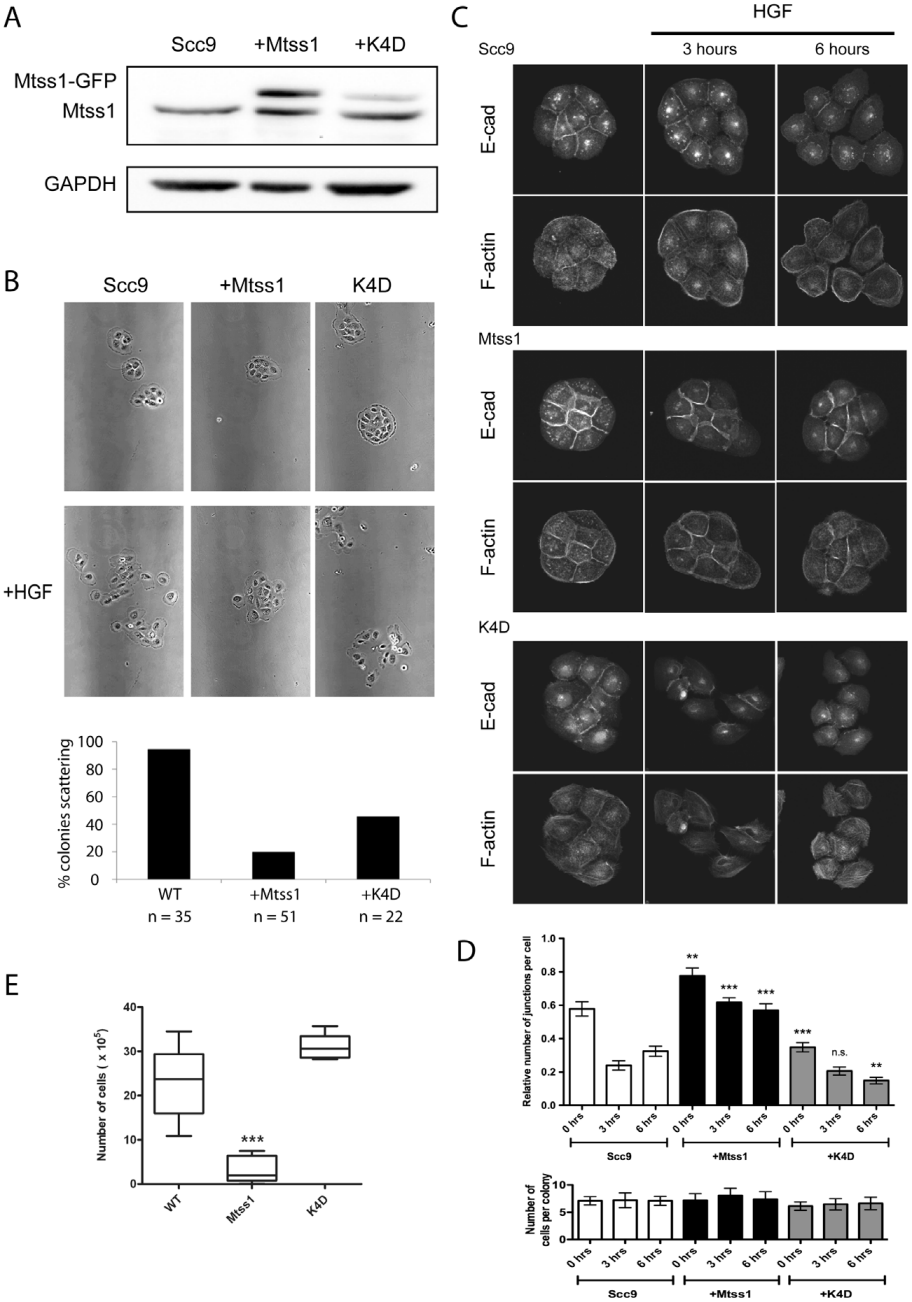
Mtss1 regulates cell-cell junction strength and inhibits HGF-scattering. (A) Mtss1 construct expression in Scc9 cells. Mtss1-GFP is approximately 3-fold over-expressed vs endogenous Mtss1 (estimated based on densitometry average from 3 experiments). (B) Small colonies of cells were incubated with 10 ng/ml HGF for 20 hours and still photos are shown from live timelapse (Movie S1). Graph is % colonies scattering n = 3 experiments. (C) Immunofluorescence labeling of E-cadherin and F-actin following HGF-induced cell scatter for 0, 3 and 6 hours in SCC9 control cells, Mtss1 expressing or K4D mutant expressing as indicated. Images representative of each time point. (D) Effect of HGF treatment on the average number of junctions labeled with E-cadherin per colony is shown relative to Scc9 cells. 40+ colonies were counted per cell line, per experiment (n = 3). (E) Number of single cells released in dispase assay assay (n = 6 experiments). For D&E Mean ± S.E.M. *** p<0.01, ** p, 0.05 by T-test.

We used HGF-induced scattering of head and neck squamous carcinoma cells (HNSCC) as a simple model for EMT to probe a role for Mtss1 as a metastatic suppressor. Stable Mtss1-GFP over-expression in Scc9 cells reduced HGF-induced scattering (Figure 1A and B; Movie S1). We also tested an inactivating four-lysine mutation of the IMD, K4D, defective in Rac and lipid binding [20]. Although we could only achieve a relatively low expression, the K4D construct only weakly inhibited HGF-induced scattering (Figure 1A and B).

HGF-induced scattering requires the breakdown of cell-cell junctions so we analyzed the localization of E-cadherin in cell colonies undergoing HGF scattering (Figure 1C). HGF treatment of Scc9 colonies reduced the number of E-cadherin cell-cell contacts by approximately half (Figure 1D). Mtss1-GFP expressing colonies were more resistant to HGF and still retained the majority of their E-cadherin junctions after 6 h (Figure 1C, D). K4D mutant expressing unstimulated colonies possessed significantly fewer cell-cell contacts compared to control cells, which underwent further disassembly in response to HGF (Figure 1C, D). Therefore the K4D mutant may be acting as a dominant negative construct that results in cell junction disassembly. It was curious that although K4D reduced the number of cell-cell contacts, it did not enhance scattering (Figure 1A) in response to HGF. This may be because expression of the K4D mutant has a slightly detrimental effect on cells overall (they grew somewhat more slowly and we were unable to express K4D to high levels, unpublished observations).

Stable cell-cell contacts contain an immobile fraction of E-cadherin, which impairs tumor cell movement (Serrels et al., 2009). We therefore hypothesized that the strengthening of cell-cell contacts by Mtss1 might lead to slower migration. Normal Scc9 cells closed scratch wounds by 20 h (Figure S1A,B Movie S2) while Mtss1-GFP expressing showed only a 15–20% decrease wound area by 20 h (Figure S1B). Furthermore, Mtss1-GFP expressing Scc9 cells still retained strong localization of E-cadherin to cell junctions (Figure S1C–E), consistent with the possibility that Mtss1 strengthens cell-cell contacts and therefore slows motility.

We also examined single cell behavior, as Mtss1 has been proposed to negatively regulate fibroblast motility [24]. Under conditions where cell-cell junctions were disassembled in confluent monolayers by overnight incubation in calcium-free medium, Mtss1-GFP expressing Scc9 cells closed wounds at a similar rate to Scc9 cells (Movie S3). Under these conditions, expression of Mtss1-GFP in SCC9 cells slightly enhances proliferation [25], so the migration defect cannot be attributed to slower proliferation. Furthermore, Mtss1-GFP expression did not affect the ability of cells plated at low density to migrate but slightly increased the rate of spreading on a mixture of collagen I and IV (Figure S2). Mtss1, thus, doesn't affect the motility of individual Scc9 cells but likely acts on cell-cell junction formation, strength and/or maintenance. Indeed, Mtss1-expressing SCC9 cells released fewer cells when mechanically disrupted in a dispase assay [26] than K4D mutant expressing or control cells (Figure 1E) suggesting increased junctional strength.

### Mtss1 localizes to cell-cell contacts

**Figure 2:**
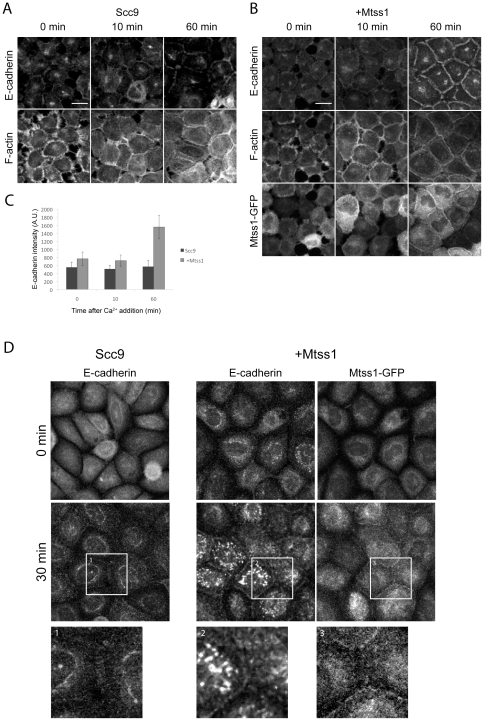
Mtss1 enhances de novo cell-cell junction formation. (A, B) Scc9 cells +/− Mtss1-GFP were treated with 2 mM EGTA for 20 min, to disrupt junctions. Reassembly was stimulated by 2 mM Ca^2+^ and cells were labeled for E-cadherin and F-actin. E-cadherin and F-actin were visualized following triton extraction and fixation to preserve the triton-insoluble junctional cytoskeleton. (A) Cell-cell junction formation in Scc9 cells and (B) Mtss1-GFP expressing Scc9 cells (C) Mean intensity of E-cadherin fluorescence at cell-cell junctions in Scc9 cells ± Mtss1 (mean ± S.D) from 3 independent experiments where n = 20 junctions. (D) Scc9 cells ± Mtss1-GFP cultured in low-Ca^2+^ KSFM overnight to disassemble adherens junctions. Cells were Ca^2+^ treated, fixed and labeled after the indicated times. Enlarged images below show boxed regions.

Mtss1 is up-regulated at the early stages of hepatocellular and basal cell carcinomas where it drives proliferation but is lost during metastatic transition [12], [13], [14], [15], [16]. Metastatic conversion during EMT [1] involves loss of cell-cell contact inhibition, frequently driven by the loss or misregulation of E-cadherin [27], [28], [29]. As Mtss1 increased the strength of cell-cell junctions, we determined the effect of Mtss1 expression on E-cadherin cell-cell junction formation (Figure 2). EGTA treatment of cells for 20 min led to a rapid departure of E-cadherin from cell-cell contact sites (0 min in Figure 2A) but when calcium was added back, Mtss1 expressing showed accelerated junctional E-cadherin recruitment (Figure 2A–C). 60 min after Ca^2+^ treatment, Mtss1 expressing cells showed more junctional E-cadherin than controls (Figure 2C). This agrees with Takaishi et al. [30], who observed that stronger cell-cell junctions contain more E-cadherin. Mtss1 is rapidly recruited (after 5 min) during cell-cell junction formation (Movie S4 and Figure 2B) and remains associated thoughout their formation in agreement with a recent report [17]. EGTA-induced cell-cell junction disassembly involves retraction followed by re-spreading of the cells. Mtss1 expression increases resistance to EGTA-induced disassembly of cell-cell junctions (data not shown) and Mtss1-GFP expressing Scc9 cells spread more readily on collagen (Figure S2). To separate spreading from cell-cell junction formation we added Ca^2+^ back to cells cultured overnight in Ca^2+^-free KSFM. Under these conditions, although there was variability within any given sample, overall we observed increased kinetics of de novo junction assembly in Mtss1-GFP expressing cells suggesting retraction/re-spreading effects were negligible (Figure 2D).

### Mtss1 depletion reduces the recruitment of F-actin to cell-cell junctions

**Figure 3:**
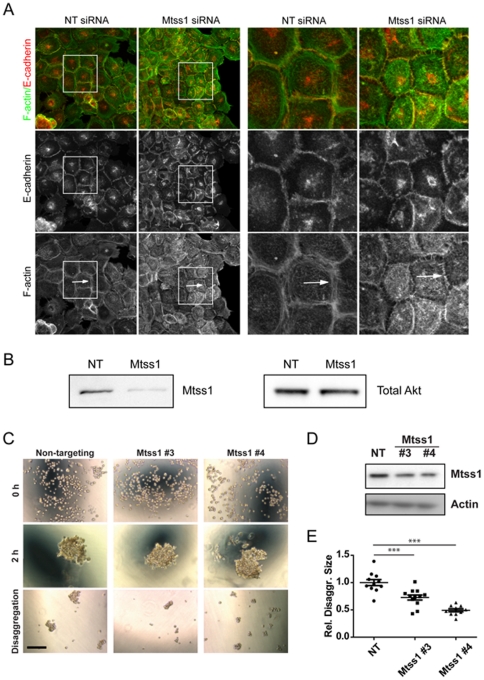
Depletion of Mtss1 affects cell-cell junction maintenance. Mtss1 siRNA disrupts cell-cell junctions in confluent Scc9 cells. (A) Scc9 cells were treated with siRNA as indicated, NT is a non-targeting siRNA. Boxed region is enlarged on the right. Arrows indicate F-actin structures observed in control cells but not Mtss1 depleted ones. (B) Western blot, representative of 3 experiments showing the Mtss1 depletion with Akt as loading control. (C–E) Normal human keratinocytes treated with siRNA oligos were aggregated in hanging drops. Phase contrast images of single-cell suspension (time zero), and aggregates before (2 h) or after trituration (disaggregation) are shown. (D) Western blot of lysates from treated cells. (E) The area of aggregates following trituration was expressed relative to the original aggregate area (2 h). Values are shown relative to scramble. *** p = 0.001, n = 2.

As Mtss1 expression accelerated cell-cell junction formation, we tested the effects of depleting Mtss1 with siRNA in confluent Scc9 cells with mature cell-cell contacts (Figure 3 A and B). Actin still appears to be recruited to junctions in Mtss1 depleted cells, but steady-state F-actin bundle structures were reduced at cell-cell contacts of Mtss1 depleted cells (Figure 3A, arrows). Mtss1 is thus required for the maintenance of actin bundles at cell-cell junctions at steady-state. Mtss1 expression (or IRSp53, IRTKS expression) induces a dramatic F-actin accumulation at the rudimentary cell-cell contacts made by fibroblastic cells [20], [21] that was not fully investigated in these previous studies. Neither ΔIMD nor K4D mutants of Mtss1 localized to or induced this phenotype, suggesting the IMD is critical for this activity [20], [21].

Mtss1 depletion didn't reduce E-cadherin junctional localization (Figure 3A), suggesting that Mtss1 functions downstream of E-cadherin to regulate junctional F-actin. Depletion of Mtss1 in primary human keratinocytes did not affect the steady state maintenance of E-cadherin at cell-cell junctions (data not shown) and cells formed aggregates comparable to controls (Figure 3C). However, depletion of Mtss1 significantly increased disaggregation due to mechanical stress, suggesting that cell-cell junctions formed in the absence of Mtss1 are weaker (Figure 3C–E). In support of our observations, MDCK cells depleted of calcium, lose E-cadherin staining from cell contact sites more slowly when they are overexpressing Mtss1 [17], but the mechanism was not explored in this study, nor the effect of Mtss1 on the strength of cell-cell contacts.

### Mtss1 drives enhanced Rac1 activation during cell-cell junction stabilization

**Figure 4:**
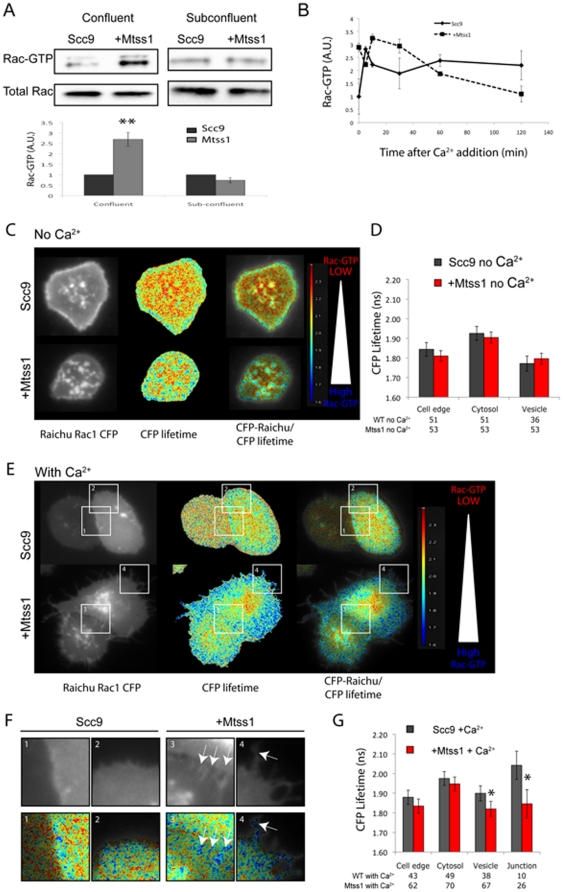
Mtss1 enhances Rac1activation during cell junction formation. (A) GST-PAK-CRIB pull down of Rac1-GTP in confluent and sub-confluent Scc9 cells ± Mtss1. Graph shows mean density (arbitrary units) of Rac1-GTP bands from confluent and sub-confluent cells normalized to total Rac1 input (n = 4 experiments. S.E.M. ** p<0.01 by t-test. (B) Scc9 cells ± Mtss1-GFP were grown to confluence and incubated with 1 mM EGTA for 60 minutes in Ca^2+^ free KSFM. After incubation with 2 mM Ca^2+^ for the indicated times, lysates Rac1-GTP was measured by G-LISA. Means from one experiment ± S.D. shown representative of 2 experiments and similar to GST-PAK CRIB pull downs (data not shown). (C) Ca^2+^ starved cells in KSFM were transfected with Raichu-Rac1 probe. CFP lifetimes are shown as a colored lookup table. Cells are in a confluent monolayer surrounded by untransfected cells. Cell edge was defined as cells not in a monolayer and not touching another cell (not shown). (D) CFP lifetime (ns) from Raichu-Rac1 probe at different parts of the cell. (E) Scc9 cells ± Mtss1 stimulated with Ca^2+^ for 30 minutes. Note cells shown are in a monolayer surrounded by untransfected cells and two Raichu-Rac1 expressing cells forming junctions are shown. (F) Enlarged regions from (E) Rac1-GTP activity at cell-cell junctions, arrows. (G) Graph is per (D) but shows lifetimes (ns) after Ca^2+^ stimulation. Mean CFP lifetime is shown from 4 independent experiments ± S.E.M in (D) and (F). * p<0.05, by one-way ANOVA.

Since Mtss1 activates Rac1 [20], [21], [22], [23], and Rac1 is a major driver of actin assembly at cell-cell contacts [7], [31], [32], [33] we hypothesized that Mtss1 could regulate actin assembly at cell-cell contacts via Rac1. Indeed Mtss1 expression drove Rac1-GTP levels up by around 2.5-fold in confluent, but not subconfluent cultures (Figure 4A). This suggests that Mtss1 activation of Rac1 is dependent on cell-cell contacts and provides a potential mechanism for how Mtss1 drives F-actin formation to maintain strong E-cadherin contacts.

Mtss1 activation of Rac1 at cell-cell contacts could be driving more rapid and stable junction formation. We thus examined the activation of Rac1 during cell junction formation (Figure 4B). EGTA treatment did not significantly change the high level of Rac1 activation in confluent Mtss1 expressing cells (Figure 4B, t = 0 minutes). Within 5 minutes after adding back calcium, Rac1-GTP transiently spiked in normal Scc9 cells, but rapidly decreased to approximately 2-fold over initial levels (Figure 4B). Rac1-GTP levels remained elevated in Mtss1-GFP expressing cells, during the initial 60 minutes of cell-cell junction formation, following which, both cell types had similar levels of Rac1-GTP.

We used the Raichu-Rac1 FRET probe tagged with a K-Ras membrane targeting sequence [34] to visualize active Rac1 during junction assembly. Raichu-Rac1 localized predominantly to the plasma membrane and internal puncta (presumably endocytic vesicles) in low Ca^2+^ KSFM of control and Mtss1 expressing cells (Figure 4C, D). After Ca^2+^ addition, most Raichu-Rac1-positive cytoplasmic puncta disappeared from control but not Mtss1-expressing cells (Figure 4E). Further, Mtss1 expressing cells showed elevated Rac1-GTP on both intracellular puncta and at cell-cell junctions (Figure 4E–G; arrows in F), consistent with our biochemical analysis (Figure 4A and B). This shows for the first time the location of active Rac in response to Mtss1 expression.

A Rab5-Rac1-Tiam1 signaling circuit has been proposed to regulate spatial Rac1 activity for signaling in cell migration [35]. Mtss1 may therefore be regulating Rac1 activity via a similar recycling mechanism involving cell-cell adhesion-regulating RacGEFs, such as Tiam1 [6], [29], [35]. Rac1 activation recruits IQGAP-1 and F-actin to cell-cell junctions, which protects E-cadherin from constitutive endocytosis and promotes junction development [9], [10]. Therefore, Mtss1-driven Rac1-GTP activity may specifically stabilize E-cadherin in cell-cell contacts by increasing F-actin and thus reducing constitutive endocytosis.

In summary, we have shown that Mtss1 regulates the activity of Rac1 during cell-cell junction formation and that Mtss1 specifically localizes Rac1-GTP to these sites. Mtss1 increases both the development and strength of cell-cell contacts, which antagonizes cell dissociation from epithelial colonies and inhibits scattering. Finally we show that Mtss1 depletion results in disruption of the actin cytoskeleton at cell-cell junctions and increases the ability of cells to disaggregate. Critically our data provide a potential mechanistic explanation for the role of Mtss1 in regulation of the metastatic potential of cancer cells through enhancing cell-cell junction strength and blocking EMT.

## Materials and Methods

### Materials

Reagents were from Sigma-Aldrich (UK) unless otherwise stated. Mtss1 was cloned into the BamH1 and EcoR1 sites of pEGFP-N1 (Clontech) and mutated using standard molecular biology methods. Raichu-Rac1 (pRaichu 1011x) was obtained from M. Matsuda (Kyoto University, Japan). Dispase was from Gibco (Invitrogen).

### Cell lines and culture

Scc9 cells were a kind gift of R. Daly (Garvan Institute, Sydney, Australia) and were maintained as described on the ATCC website. Stable Scc9 cells expressing Mtss1-GFP were selected in 0.5 mg/ml G418. For Ca^2+^ chelation experiments, cells were incubated with 2 mM EGTA in Ca^2+^-free DMEM (Invitrogen) or were incubated overnight in KSFM-Ca^2+^ free medium (Invitrogen). 2 mM CaCl_2_ was added to induce junctions.

Scc9 cells were transfected using Polyfect (Qiagen) for DNA and Hyperfect (Qiagen) for siRNA using the manufacturer's protocol. Normal keratinocytes were transfected using Interferin (PeqLab) according to the manufacturer's protocol. Mtss1 siRNA was from Qiagen (Hs_Mtss1_5, target sequence, 5′-CCGACGGATGTTCCAAGCCAA-3′) or Dharmacon (Mtss1 #3 5′-CCAGUUGUCUAACGGGUUU-3′, Mtss1 #4 5′-CAAGUGAACAGGUGAUUCU-3′). All stars-negative control or NT oligo controls were from Qiagen (1027280) and Dharmacon (D-00210-02).

For HGF-induced scattering, cells were seeded in 6-well plates at 0.5×10^5^ cells/well and cultured for 2–3 days. Cells were stimulated with 10 ng/ml HGF for 20 h.

The dispase assay was performed as described in [26]. Briefly, cells were grown to confluence over 3 days, washed with PBS and incubated with 2 mg/ml dispase/PBS (Gibco, Invitrogen) Monolayers were mechanically dissociated with a P1000 Gilson Pipetman followed by a 70 µm filter.

Normal keratinocytes were grown as described previously (Braga et al., 1997). For aggregation assays, normal keratinocytes were trypsinised in 500 µl aggregation assay buffer (60% versene (v/v), 0.1% trypsin, 0.1 mM CaCl_2_) to protect cadherin receptors from cleavage until single-cell suspension. Cells were counted and centrifuged at 900 rpm for 5 minutes. The cell pellet was resuspended in standard calcium medium (5×10^4^cells/ml), set as hanging drops in a humid chamber (6 droplets per sample) and incubated for up to 120 minutes. Cells were pipetted gently to disaggregate clusters. Images were taken on an Olympus CKX41 microscope with a Colour View IIIu camera using cell̂D v. 2.4 software (Olympus). Areas of all aggregates following trituration were quantified using ImageJ and normalised to the initial aggregate in each droplet. Control values were arbitrarily set as 1 and Mtss1-depleted cell data expressed relative to controls.

### FLIM-FRET analysis

Cells in KSFM-Ca^2+^ free medium were transfected with Raichu-Rac1 and imaged the following day. Fluorescence resonance energy transfer (FRET) was detected using a Lambert Instruments fluorescence attachment (LIFA) on a Nikon Eclipse TE 2000-U microscope equipped with a 100× oil immersion objective and a filter block consisting of a 436/20× excitation filter, a T455LP dichroic mirror, and a 480/40 M emission filter. We used a modulated 445 nm LED as light source, which, in combination with the modulated intensifier from the LIFA system. A FITC solution was used as a reference. Donor lifetime was analyzed using the Fluorescence Lifetime Imaging Microscopy (FLIM) software (version 1.2.7; Lambert Instruments, The Netherlands). For analysis, a 4×4 pixel box was used to measure the average lifetime from five different areas of the indicated regions, in a given cell. These measurements were used to calculate an average lifetime of that region in that cell.

### Immunofluorescence

Cells were fixed in either 4% paraformaldehyde, followed by permeablisation in 0.1% Triton X-100 or for visualisation of E-cadherin junctions, fixed in 4% paraformaldehyde containing 0.1% Triton X-100 for 10 minutes which results in a greater extraction of Triton X-100 soluble proteins. Cells were labelled with mouse anti-E-cadherin (clone 36/E-Cadherin, BD Biosciences) diluted 1/500 and Alexa-350 phalloidin (Invitrogen). Images were acquired using an Olympus FV1000 confocal microscope and a 60× objective. Z-stacks were acquired at 0.5 µm intervals. Images were analyzed in ImageJ.

### Rac activation assays and Western blotting

Rac-GTP pulldowns were performed as described [21] and [20]. 500 µg of cell lysates were incubated with 5 µl PAK-CRIB beads (approximately 20 mg) for 1 hour. Beads were then washed with lysis buffer, boiled and resolved on SDS-PAGE (4–12% gels; Invitrogen) before western blotting. Blots were probed with rabbit anti-Mtss1 [20], and mouse anti-Rac (23A8, Upstate). Rac-GTP levels were quantified using imageJ and normalized to total Rac expression. Rac-GLISA was performed following the manufacturer's instructions (Cytoskelton Inc., USA).

### Analysis of random cell motility and spreading

6-well plates were coated with a mixture of collage I and IV (at 10 µg/ml for 1 hour) and cells were seeded overnight. The number of phase dark cells was counted as a measure of cell spreading. For cell motility, cells were seeded overnight and then imaged using a Nikon TE2000 microscope. Images were acquired every 15 minutes for 24 hours. Random cell motility was measured using the manual tracking plug-in and data analyzed using the Ibidi chemotaxis and migration tool plug-in for ImageJ.

## Supporting Information

Figure S1 **Mtss1 inhibits collective motility in a scratch wound assay.** (A) Scc9 cells and Mtss1-GFP expressing cells were grown to confluence and scratched using a pipette tip. Wound healing was observed using time-lapse microscopy over 20 hours (see Movie S2). (B) Quantification of Mtss1 inhibition of scratch wound migration. Area of the wound at different time points was calculated in ImageJ and data is shown relative to time = 0 hrs. Mean ± S.E.M. is shown from 4 independent experiments. (C) Scc9 cells or Mtss1-GFP expressing Scc9 cells were grown to confluence on coverslips and wounded using a yellow pipette tip. Cells were fixed 2 hours after wounding and labeled for E-cadherin and F-actin. Cross sections are show for the regions indicated by a white line (C) are shown in (D). (E) Quantification of E-cadherin positive cell-cell junctions on the wound edge. Mean ± S.D. from two experiments is shown.
(TIF)

Figure S2 **Mtss1 enhances cell spreading on 2D surfaces, but does not affect random single cell motility.** Mtss1-GFP expressing Scc9 cells and Scc9 cells alone were seeded into 6-well plates on a mixture of collagen I/IV (total of 10 mg protein). Cells were allowed to adhere over 3 hours to assess their ability to spread (A). (B) Images were acquired every hour and the number of cells that had spread (phase dark cells) were counted. Graph shows average percentage of spread cells at the times indicated ± S.E.M. from three independent experiments. T-test was performed vs Scc9 cells and p values are indicated on the graph. (C) The random migration of Mtss1-GFP Scc9 cells and Scc9 cells alone was tested on collagen I/IV over 20 hours. Graph represents total distance traveled and velocity relative to Scc9 cells alone. Mean is shown ± S.D. from two experiments.
(TIF)

Movie S1 **Mtss1 inhibits HGF-induced scattering.** Sub-confluent Scc9 cells were grown for 2 days to allow colony formation and then HGF was added (10 ng/ml) for 20 hours. Scc9 cells (left panel), Mtss1-GFP (middle panel) or Mtss1-K4D-GFP (right panel) expressing Scc9 cells are shown.
(MOV)

Movie S2 **Mtss1 slows scratch would induced motility.** Scc9 cells (left panel) and Mtss1-GFP expressing Scc9 cells (right panel) were grown to confluence and wounded with a pipette tip and wound closure was followed over 24 hours.
(MOV)

Movie S3 **Mtss1 has no effect on scratch wound motility of calcium depleted cells.** Scc9 cells (left panel) and Mtss1-GFP expressing Scc9 cells (right panel) were grown to confluence and switched to Ca^2+^-free KSFM overnight. Cells were then wounded with a pipette tip and wound closure was followed over 24 hours.
(MOV)

Movie S4 **Mtss1 localizes to zipper-like structures at cell-cell junctions.** Mtss1-GFP expressing Scc9 cells were treated with 2 mM EGTA for 20 minutes to disassemble cell-cell junctions. Cells were washed with complete medium and Mtss1-GFP and F-actin (mCherry-lifeact) imaged over the following hour to visualize the reformation of cell-cell junctions. Mtss1-GFP localizes to cell-cell junctions following the initial cell-cell contact and co-localizes with F-actin. Left panel shows Mtss1-GFP and the right panel shows mCherry-lifeact. Images were acquired every 5 minutes.
(MOV)
