# Supplementary figures and images for: Mtss1 Promotes Cell-Cell Junction Assembly and Stability through the Small GTPase Rac1
Source: PLoS One. 2012 Mar 27;7(3):e31141. doi: 10.1371/journal.pone.0031141 (PMC3313965; doi:10.1371/journal.pone.0031141)

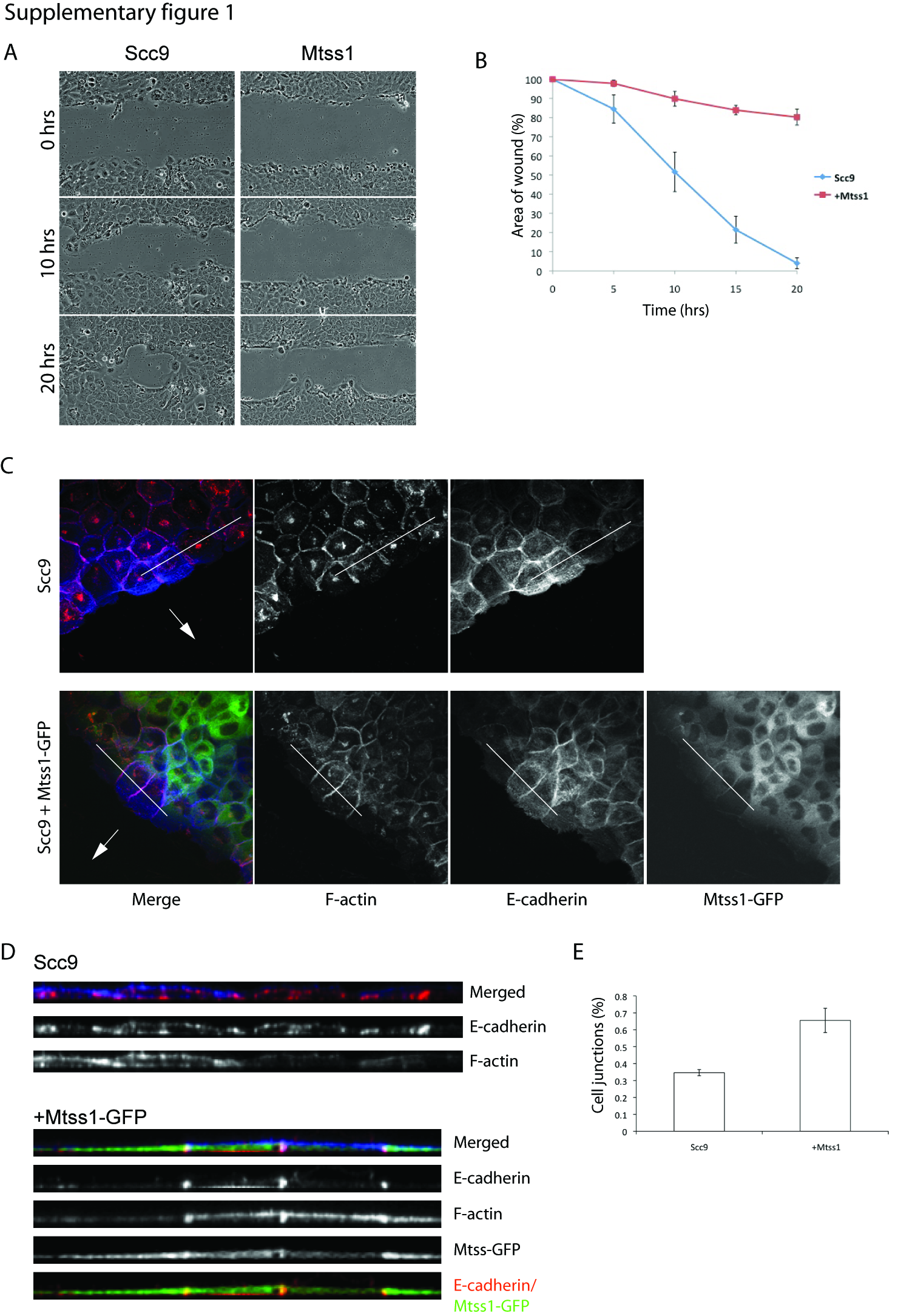

Supplement: Figure S1 — Mtss1 inhibits collective motility in a scratch wound assay. (A) Scc9 cells and Mtss1-GFP expressing cells were grown to confluence and scratched using a pipette tip. Wound healing was observed using time-lapse microscopy over 20 hours (see Movie S2). (B) Quantification of Mtss1 inhibition of scratch wound migration. Area of the wound at different time points was calculated in ImageJ and data is shown relative to time = 0 hrs. Mean ± S.E.M. is shown from 4 independent experiments. (C) Scc9 cells or Mtss1-GFP expressing Scc9 cells were grown to confluence on coverslips and wounded using a yellow pipette tip. Cells were fixed 2 hours after wounding and labeled for E-cadherin and F-actin. Cross sections are show for the regions indicated by a white line (C) are shown in (D). (E) Quantification of E-cadherin positive cell-cell junctions on the wound edge. Mean ± S.D. from two experiments is shown. (TIF) [file pone.0031141.s001.tif]

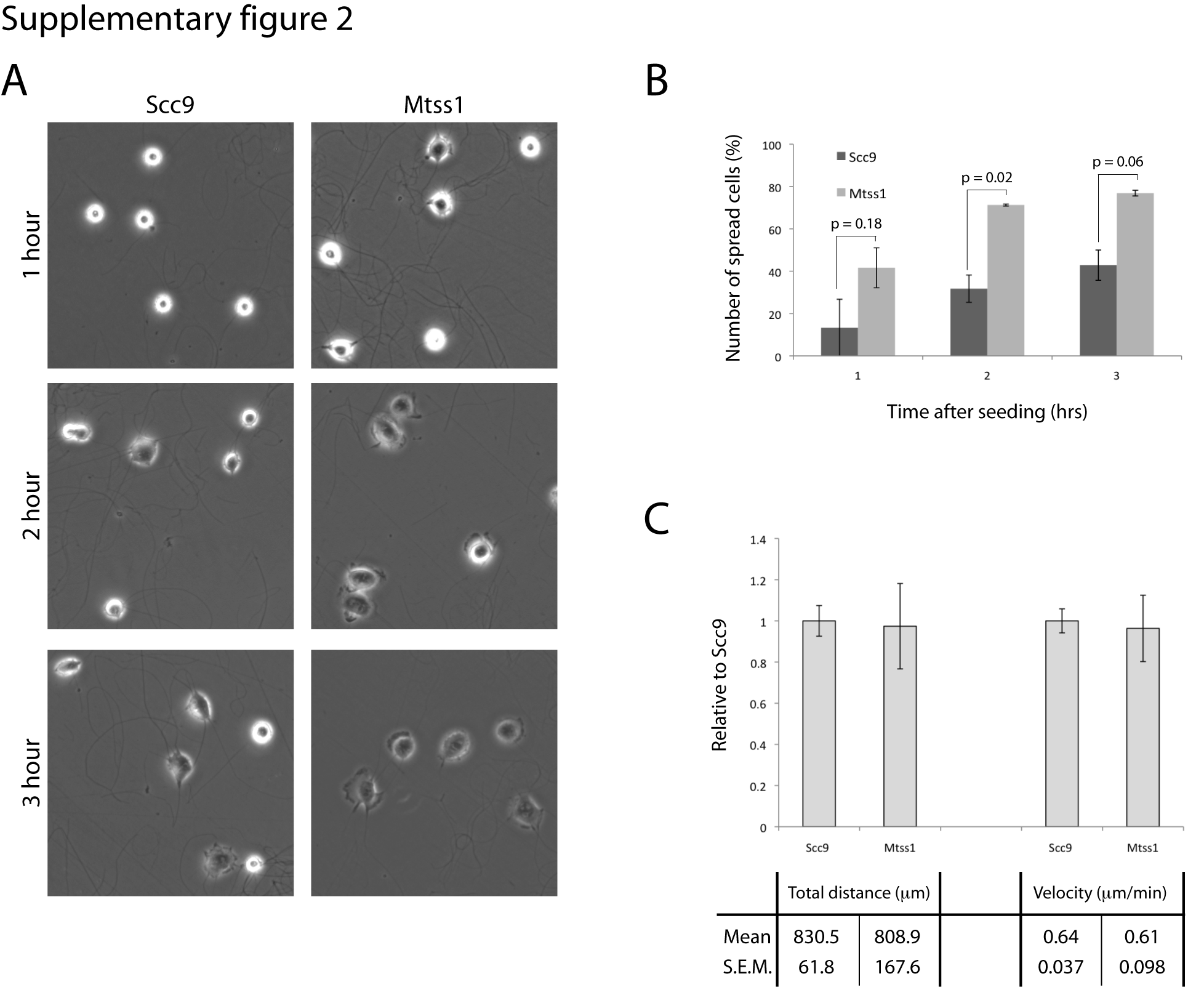

Supplement: Figure S2 — Mtss1 enhances cell spreading on 2D surfaces, but does not affect random single cell motility. Mtss1-GFP expressing Scc9 cells and Scc9 cells alone were seeded into 6-well plates on a mixture of collagen I/IV (total of 10 mg protein). Cells were allowed to adhere over 3 hours to assess their ability to spread (A). (B) Images were acquired every hour and the number of cells that had spread (phase dark cells) were counted. Graph shows average percentage of spread cells at the times indicated ± S.E.M. from three independent experiments. T-test was performed vs Scc9 cells and p values are indicated on the graph. (C) The random migration of Mtss1-GFP Scc9 cells and Scc9 cells alone was tested on collagen I/IV over 20 hours. Graph represents total distance traveled and velocity relative to Scc9 cells alone. Mean is shown ± S.D. from two experiments. (TIF) [file pone.0031141.s002.tif]
